# Freestanding lipid bilayer tensiometer for the study of mechanosensitive ion channels

**DOI:** 10.1073/pnas.2221541120

**Published:** 2023-03-13

**Authors:** Gonzalo Pérez-Mitta, Roderick MacKinnon

**Affiliations:** ^a^HHMI, The Rockefeller University, New York, NY, 10065; ^b^Laboratory of Molecular Neurobiology and Biophysics, The Rockefeller University, New York, NY, 10065

**Keywords:** mechanosensitive ion channel, lipid bilayer, tension measurement, TRAAK

## Abstract

Mechanical forces modify the cell membrane potential by opening mechanosensitive ion channels. We report the design and construction of a lipid bilayer tensiometer to study channels that respond to lateral membrane tension, γ , in the range 0.2 to 1.4 $\frac{k_{B}T}{{\mathrm{nm}}^{2}}$ (0.8 to 5.7 $\frac{\mathrm{mN}}{m}$ ). The instrument consists of a black-lipid-membrane bilayer, a custom-built microscope, and a high-resolution manometer. Values of γ are obtained from the determination of the bilayer curvature as a function of applied pressure by means of the Young–Laplace equation. We demonstrate that γ can be determined by calculating the bilayer radius of curvature from fluorescence microscopy imaging or from measurements of the bilayer’s electrical capacitance, both yielding similar results. Using electrical capacitance, we show that the mechanosensitive potassium channel TRAAK responds to γ , not curvature. TRAAK channel open probability increases as γ is increased from 0.2 to 1.4 $\frac{k_{B}T}{{\mathrm{nm}}^{2}}$ but open probability never reaches 0.5. Thus, TRAAK opens over a wide range of γ , but with a tension sensitivity about one-fifth that of the bacterial mechanosensitive channel MscL.

## Significance

Stretch-activated ion channels respond to tension in cellular membranes, transducing mechanical stimuli into physiological responses, including the sense of touch and blood pressure regulation. However, the precise measurement of tensions required to activate mechanosensitive ion channels is lacking, since techniques thus far have relied on less accurate determinations of shear force or pipette pressure. This study presents the design, fabrication, and characterization of an instrument to assess simultaneously ion-channel current and lateral membrane tension. We show that the system can control tensions spanning a range of values relevant to most stretch-activated channels. Using the mammalian ion channel TRAAK, we construct tension activation curves and determine its tension sensitivity.

The action of mechanically activated ion channels mediates fast mechanosensation in all domains of life (1, 2). There are different ways in which forces can be coupled to mechanosensitive channels to modulate their gating. Some channels may be activated by forces transmitted through molecular tethers, while others respond directly to lateral membrane tension (3, 4). The latter include the prokaryotic channels MscL and MscS, the eukaryotic K2P K^+^ channels TRAAK and TREK, and the Piezo channels (5–9). Because these channels respond to lateral membrane tension as a primary gating stimulus, we built a device to control lateral membrane tension in the membrane of reconstituted ion channels.

Commonly used methods to apply a mechanical stimulus include poking the cell surface and pressurization of gigaseal patches, where the control variables are the poking displacement of a probe or the pressure in a pipette relative to atmospheric pressure (10, 11). Cell poking has the advantage of operating on channels in their native environment of an intact cell membrane. The disadvantage is that probe displacement is not convertible to a quantity relevant to forces on the molecular scale. Patch pressurization experiments have permitted the construction of tension activation curves (12, 13), but the accuracy of these is limited for two reasons. First, it is difficult to measure accurately the curvature of small membrane patches looking through a glass capillary. Second, membrane patches attached to the capillary typically do not remain stationary, causing the patch to move uncontrollably and the tension to change over time, even in the absence of applied pressure (14, 15).

Accurate measurements of membrane tension have been made on pure lipid membranes using giant unilamellar vesicle (GUV) aspiration pipettes and atomic force microscopy (AFM) (16, 17). However, these methods are not easily combined with electrical recordings of ion channels. Existing configurations of AFM are not compatible with electrophysiological recordings, and even though whole GUV patching is a promising approach, it has not been successfully applied to ion-channel research. The main reason underlying this failure so far is that to measure lateral membrane tension and ion-channel currents on a GUV, one needs to find a balance between a good electrical seal and preventing the adhesion of lipids to the patch pipette (18). This has proven impossible for lipid membranes containing ion channels, leading to large nonspecific (leak) currents.

We have taken the conceptually simple approach of controlling the pressure across freestanding lipid bilayers while measuring optically the membrane curvature to calculate lateral membrane tension. We then interrogate under what conditions membrane electrical capacitance permits the calculation of membrane area so that capacitance can be used as a surrogate for optical area measurement. Two regimes of membrane pressurization, equilibrium and nonequilibrium, permitted us to estimate zero-pressure membrane tension and determine higher applied tensions, respectively. This way, we could determine tensions within a range of 0.2 to 1.4 $\frac{k_{B}T}{{\mathrm{nm}}^{2}}$ . We applied the method to analyze the tension dependence of TRAAK K^+^ channel gating. TRAAK is a mechanosensitive channel involved in mechanical nociception whose tension dependence has not been studied quantitatively (19). Under our experimental conditions, we find that TRAAK is open with a low open probability at zero pressure tensions (0.2 to 0.3 $\frac{k_{B}T}{{\mathrm{nm}}^{2}}$ ) and opens further with a weak dependence on tension, not leveling off even for the highest tensions applied. TRAAK’s shallow responsiveness over a wide tension range distinguishes it from other mechanosensitive channels like MscL and Piezo1.

## Results

### Lateral Tension of Freestanding Bilayers at Equilibrium.

To calculate the tension of freestanding bilayers, we use the Young–Laplace equation

**Fig. 1.**
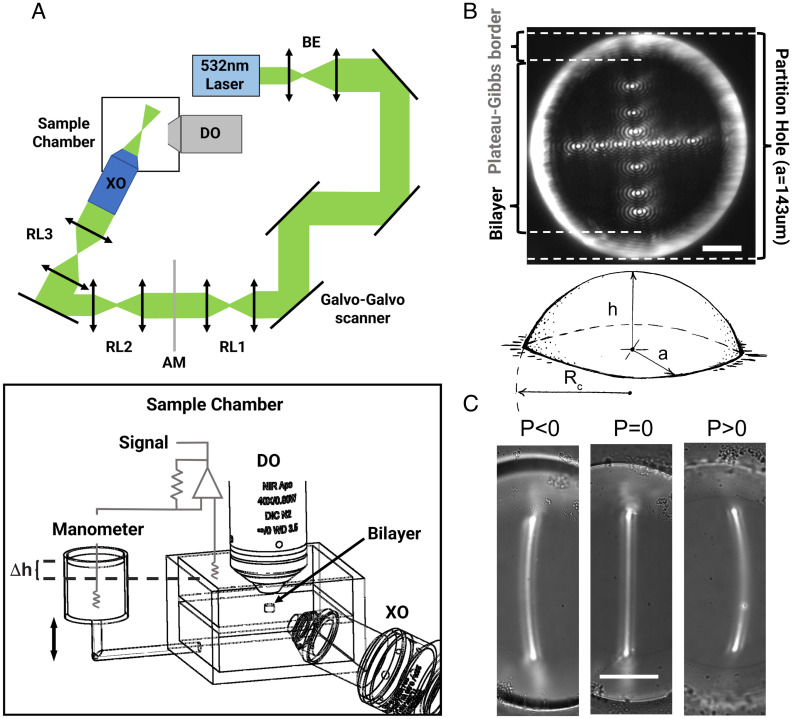
Apparatus design and imaging experiments. (*A*, *Top*) Scheme of the illumination optics for the bilayer microscope. Components are: a 532-nm laser, 2× beam expander (BE), galvo-galvo scanner, three relay lenses (RL1-3), an apodization mask (AM), and detection and excitation objectives, DO and XO, respectively. (*Bottom*) a schematic drawing of the recording chamber that shows the main components required for these studies: a manometer, voltage-clamp amplifier, and detection and excitation objectives. A black arrow points to the position of the lipid bilayer relative to the objectives. (*B*, *Top*) fluorescence image of a DOPE:POPC (2:1 wt:wt) lipid bilayer with Liss-rhodamine PE (10^−4^ wt:wt) showing the cross-like array used to map the shape of a freestanding bilayer in space. Both the position of the bilayer and the surrounding torus (Plateau–Gibbs border) are indicated. (Scale bar: 50 µm.) (*Bottom*) diagram depicting the main geometric parameters of a curved bilayer: central displacement ( h ), the radius of the hole or bilayer's perimeter ( a ), and radius of curvature ( $R_{c}$ ). (*C*) Zero-pressure finding sequence: Images were taken on a DOPE:POPC 2:1 + Liss rhodamine PE (10^−4^ wt:wt) lipid bilayer imaged by scanning a focused laser laterally at different pressures. The projection of the intersection between the light sheet and bilayer can be seen at the center of each image, curved for P ≠ 0 and straight for P = 0. (Scale bar: 30 µm.)

[1] $$\Delta P=\gamma \left(\frac{1}{R_{c_{1}}},+,\frac{1}{R_{c_{2}}}\right),$$

which relates the mean curvature, $\frac{1}{2}\left(\frac{1}{R_{c1}}+\frac{1}{R_{c2}}\right)$ , $R_{c1}$ and $R_{c2}$ being the principal radii of curvature (inverse of principal curvatures), to the pressure difference across the membrane bilayer, ΔP . The proportionality constant, γ , is the lateral membrane tension of the free bilayer^*^. Thus, by measuring the geometry of the bilayer and the pressure difference across it, the lateral membrane tension can be determined. The measurements were made using a sample chamber consisting of two aqueous reservoirs (top and bottom) separated by a plastic partition. Each partition contained a ~100 µm diameter hole where bilayers were formed from lipid solutions in n-decane. Pressures were applied using a manometer based on a computer-controlled micromanipulator that lifted a water vessel to different heights relative to the water level of the top chamber (Fig. 1*A*). Pressure values were then measured as differences in water level in $mmH_{2}O$.

The radii of curvature were measured using a widefield fluorescence microscope adapted to image freestanding bilayers doped with a small amount of fluorescently labeled lipid (~10^−4^ wt:wt) (Fig. 1*B*). The detection optics consisted of a commercial upright electrophysiology microscope coupled to a scientific Complementary Metal-Oxide-Semiconductor (sCMOS) camera with appropriate filters. The illumination optics consisted of a diode-pumped green laser (532 nm) expanded and fed into a galvo-galvo scanner that steered the beam horizontally and vertically. The scanned beam was then illuminated onto an apodization mask (AM) that shaped it into a ring that, upon focusing through the excitation objective (XO), formed a Bessel beam at the bilayer (focal) plane (20). The optical planes of the Galvo scanner, AM, and XO were conjugated by appropriate relay lenses. The focused Bessel beam allowed localization of any point on the bilayer with an accuracy of fewer than 3 μm in both lateral and axial directions.

Before starting each experiment, the beam was scanned laterally to find the zero-pressure-difference point, as shown (Fig. 1*C*). The idea behind this method is that when the pressure difference is zero, the projected intersection between the scanned beam and the bilayer is a line (intersection of two planes). By contrast, the bilayer will have a nonzero curvature for pressures different than zero, and the projection will be a curve (intersection of a plane and a sphere). This method permitted us to define the zero-pressure point within 200 µm of H_2_O and then define all pressure differences relative to it.

**Fig. 2.**
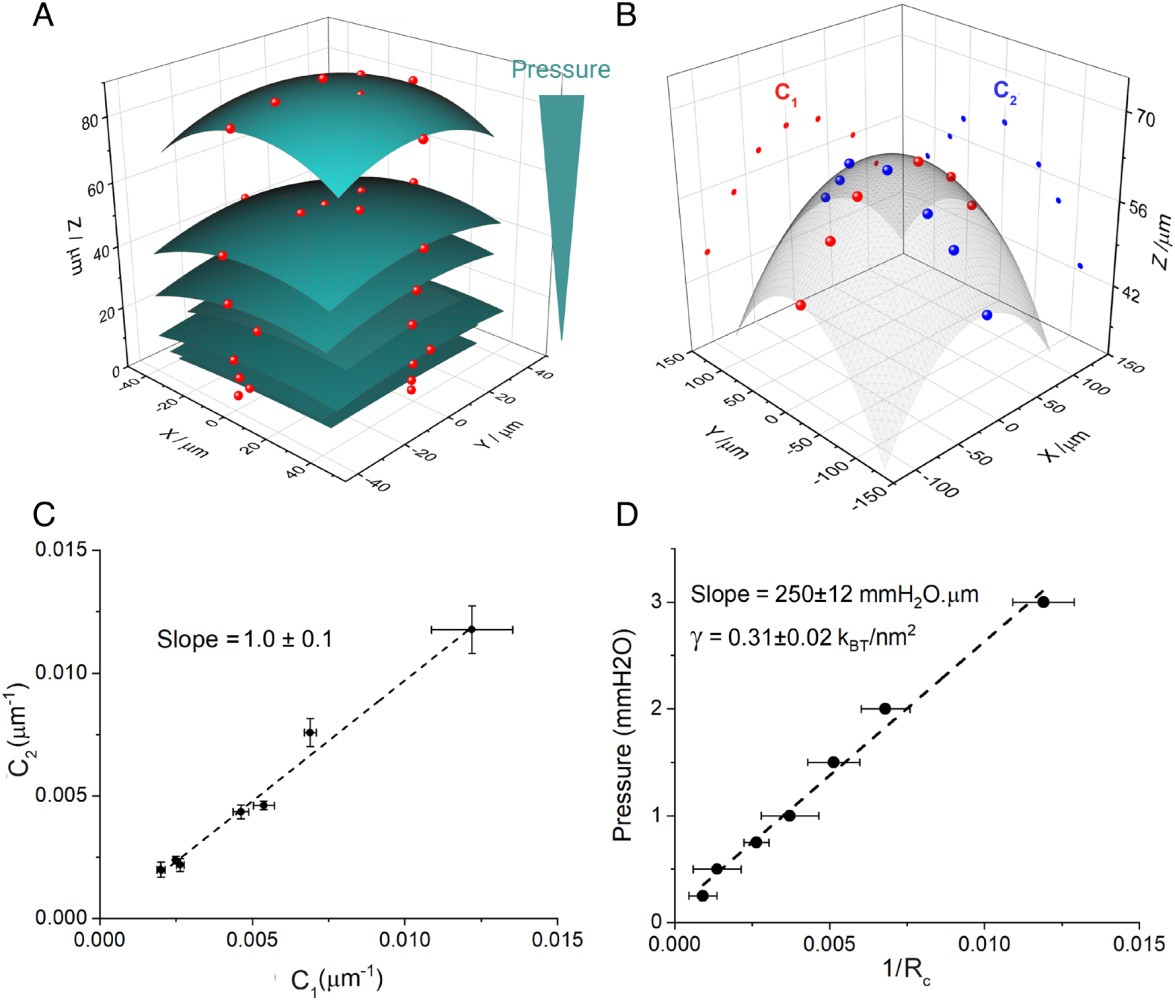
Measurements in equilibrium. Optical. (*A*) Points obtained from the illumination array shown in Fig. 1*B* mapped in space for increasing pressures (red points) and their respective spherical fit (green surfaces). (*B*) Replotting of the points at the highest pressure on panel *A* and projection on the XZ (red) and YZ (blue) planes. Principal curvatures $C_{1}$ (red) and $C_{2}$ (blue) were obtained from circular fits on the projected data. Spherical fit shown as a gray surface. (*C*) Plot of the relation between the principal curvatures, $C_{2}$ vs. $C_{1}$ , for 7 different bilayers under pressure. The slope of 1.0 ± 0.1 indicates that bilayers bulge as spherical caps ( $C_{2}=C_{1}$ ). (*D*) Application of the Young–Laplace equation: Pressure vs. curvature ( $\frac{1}{R_{c}}$ ) for the bilayer shown in A, the slope (2X bilayer tension, γ ) is 250 ± 10 $mmH_{2}O.\mu m$ (0.31 ± 0.02 $\frac{k_{B}T}{{\mathrm{nm}}^{2}}$).

To measure the principal curvatures of the bilayer, we used an array of 13 points that were positioned on the bilayer using the galvo scanner (Fig. 1*B*). XY coordinates were obtained by direct mapping of the images, while Z coordinates were obtained from the displacement of the focal point measured with a linear gauge coupled to the microscope. Each point was thus mapped in 3 dimensions, reconstructing the shape of the bilayer for each pressure (Fig. 2*A*). From these reconstructions, we measured the principal curvatures ( $C_{2}$ and $C_{1}$ ) of a given bilayer under pressure by projecting the points on the XZ and YZ planes and fitting each to the arc of a circle (Fig. 2*B*). By plotting $C_{2}$ vs. $C_{1}$ for different bilayers under enough pressure to significantly curve the bilayers (1 to 3 $mmH_{2}O$ ), we found that the slope of the curve was close to 1.0, thus $C_{1}$ = $C_{2}$ , defining a spherical surface (Fig. 2*C*). Eq. **1** therefore becomes

[2] $$\Delta P=2\;\gamma \left(\frac{1}{R_{c}}\right).$$

with $R_{c}$ being the radius of the spherical surface. The array of points for each bilayer was fit to a spherical cap (Fig. 1*B*) to estimate $R_{c}$ . Plotting applied pressure as a function of $\frac{1}{R_{C}}$ yielded an approximately straight line with a slope, according to Eq. **2**, equal to 2 γ (Fig. 2*D*).

One caveat to the procedure discussed so far is that it takes ~1 min to map the bilayer geometry for a given pressure. For reasons that will become clear later in the paper, to study channels we needed a faster measurement of the bilayer curvature, ideally on the order of 10 to 100 ms. We reasoned that if we could use electrical capacitance to estimate the bilayer area A (see below), then knowing the bilayer hole radius a (Fig. 1*B*), obtainable through a single microscopic image, would allow us to calculate $R_{c}$ through the relationship (21),

[3] $$R_{c}=\frac{A}{\sqrt{4\;A\;\pi -4\;a^{2}\pi^{2}}}.$$

**Fig. 3.**
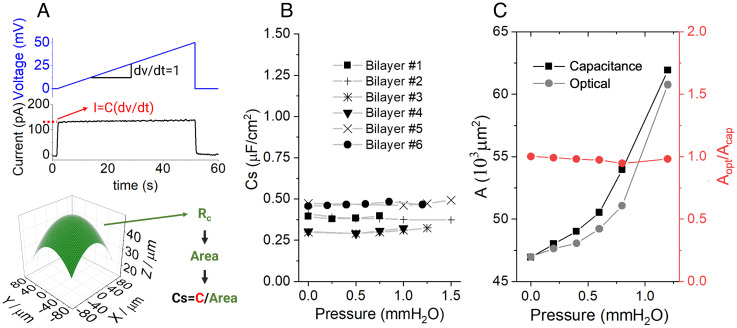
Measurements in equilibrium. Capacitance. (*A*, *Top*) voltage protocol (blue) applied to obtain the capacitance (C) from the measured current (black). The relationship between current (I) and capacitance (C) is depicted in red. Immediately after the voltage ramp starts ( dV/dt=1 ), the capacitance can be directly read from the current, as indicated by the red dotted line. *Bottom:* Diagram depicting the procedure for calculating the specific capacitance (Cs) of a bilayer. The area of the bilayer is calculated from the radius of curvature (R_c_) obtained as described in Fig. 2. Then, the specific capacitance (Cs) is calculated as the ratio between the capacitance (C) and the area at every pressure. (*B*) Cs as a function of pressure for 6 different bilayers. (*C*) Comparison between optical and capacitance methods. Left axis: Area calculated optically (grey circles) and from capacitance (black squares) for a 1,2-dioleoyl-sn-glycero-3-phosphoethanolamine (DOPE):1-palmitoyl-2-oleoyl-sn-glycero-3-phosphocholine (POPC) (2:1) bilayer as a function of pressure. Right axis: The ratio between the areas calculated optically and from capacitance (A_opt_/A_cap_) as a function of pressure.

The basic idea behind this approach is that a bilayer is a parallel plate capacitor whose capacitance is inversely proportional to thickness and directly proportional to area (22). Cell membranes exhibit a reasonably constant capacitance per area, called specific capacitance (Cs) (23), but in our case, the bilayer contains a torus of decane and lipids surrounding its perimeter. For this reason, we needed to test whether specific capacitance remains constant when the area changes during bilayer pressurization. We determined capacitance by applying a voltage ramp as shown (Fig. 3*A*) while changing pressure across the bilayer. At the same time, we measured the area of the bilayer optically as described (Fig. 2). Fig. 3*B* graphs the capacitance divided by measured area, i.e., the specific capacitance, for six bilayers. From this graph, we reached two conclusions. First and most importantly, the specific capacitance for a given bilayer remained constant (within ~10%) during pressurization. Second, the specific capacitance among bilayers ranged from 0.3 to 0.5 $\frac{\mu F}{{\mathrm{cm}}^{2}}$ . These observations imply that a single measurement of specific capacitance in an unpressurized bilayer can be used to convert capacitance to area. Fig. 3*C* shows for the same bilayer being pressurized, the area measured both optically and with capacitance. The capacitance method, which is much faster, yields a reasonable estimate of membrane area. We emphasize that the validity of the approach requires one measurement of specific capacitance for each bilayer, for example, at zero pressure, because the value, while approximately constant during pressurization, is not the same for all bilayers. We also note that it is important to use partitions with round holes for this method given its reliance on spherical geometry.

**Fig. 4.**
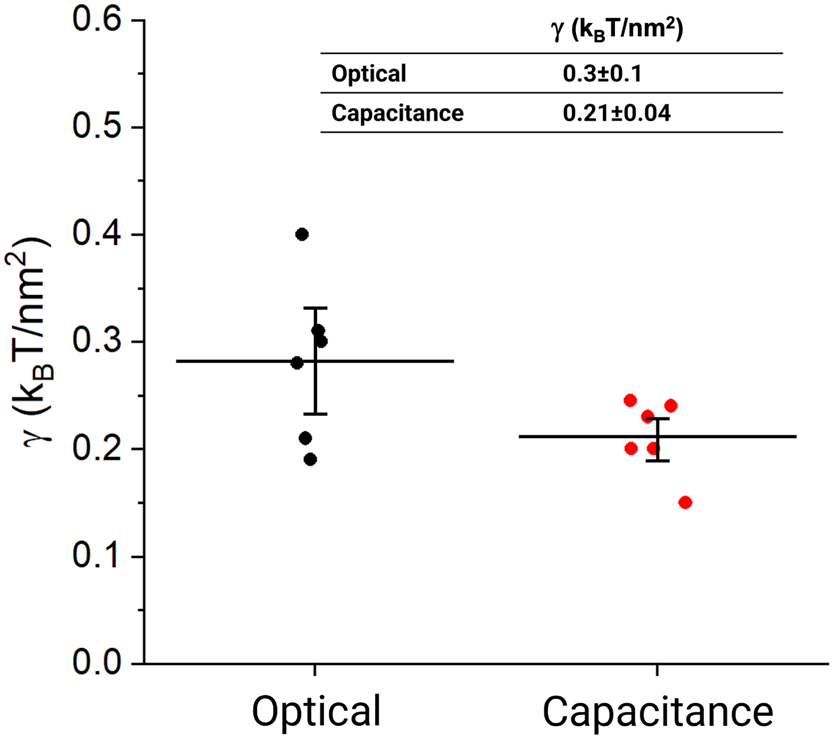
Measurements in equilibrium. Comparison of calculated tensions from optical and capacitance methods. Bilayer tension values for different bilayers (filled circles) were calculated from optical (n = 6, black) and capacitance (n = 6, red) measurements.

When we determined the tension of bilayers by graphing pressure as a function of $\frac{1}{R_{c}}$ , as in Fig. 2*D*, we obtained a similar result, 0.2 to 0.3 $\frac{k_{B}T}{{\mathrm{nm}}^{2}}$ , whether $R_{c}$ was obtained using the optical or capacitance method (Fig. 4). Since the two methods gave consistent results that agree with past estimates of lateral tension in free bilayers (24), and since the capacitance method is rapid, we used it for what comes next.

To motivate what comes next, we raise a question that is undoubtedly on the minds of many readers. Why, when we increase the pressure, does the bilayer tension remain constant (i.e., why are the data in Fig. 2*D* linear)? The key word in the heading to this section of the paper is “equilibrium”. Relatively small pressures have been applied slowly, allowing lipids from the torus to enter the bilayer and increase its area. In other words, as ΔP is increased, $R_{c}$ adjusts and γ returns to its starting value. Ultimately, at equilibrium, γ is determined by the chemical interaction between the membrane/torus with the partition material. By this reasoning, the tension range 0.2 to 0.3 $\frac{k_{B}T}{{\mathrm{nm}}^{2}}$ approximates the unpressurized tension of the free bilayer. Ultimately, we wish to know how mechanosensitive ion channels change their gating in response to changes in lateral membrane tension. This becomes the focus of the following experiments.

### Lateral Tension out of Equilibrium.

The torus of a freestanding bilayer is shaped axially like a wedge from the resulting interfacial forces between the decane, lipid, water, the partition material, and a zero-degree contact angle imposed by the planar bilayer (*SI Appendix*, Fig. S1). At equilibrium, this multicomponent phase dominates the chemical and mechanical properties of the bilayer and keeps its tension constant, as shown in the previous section.

**Fig. 5.**
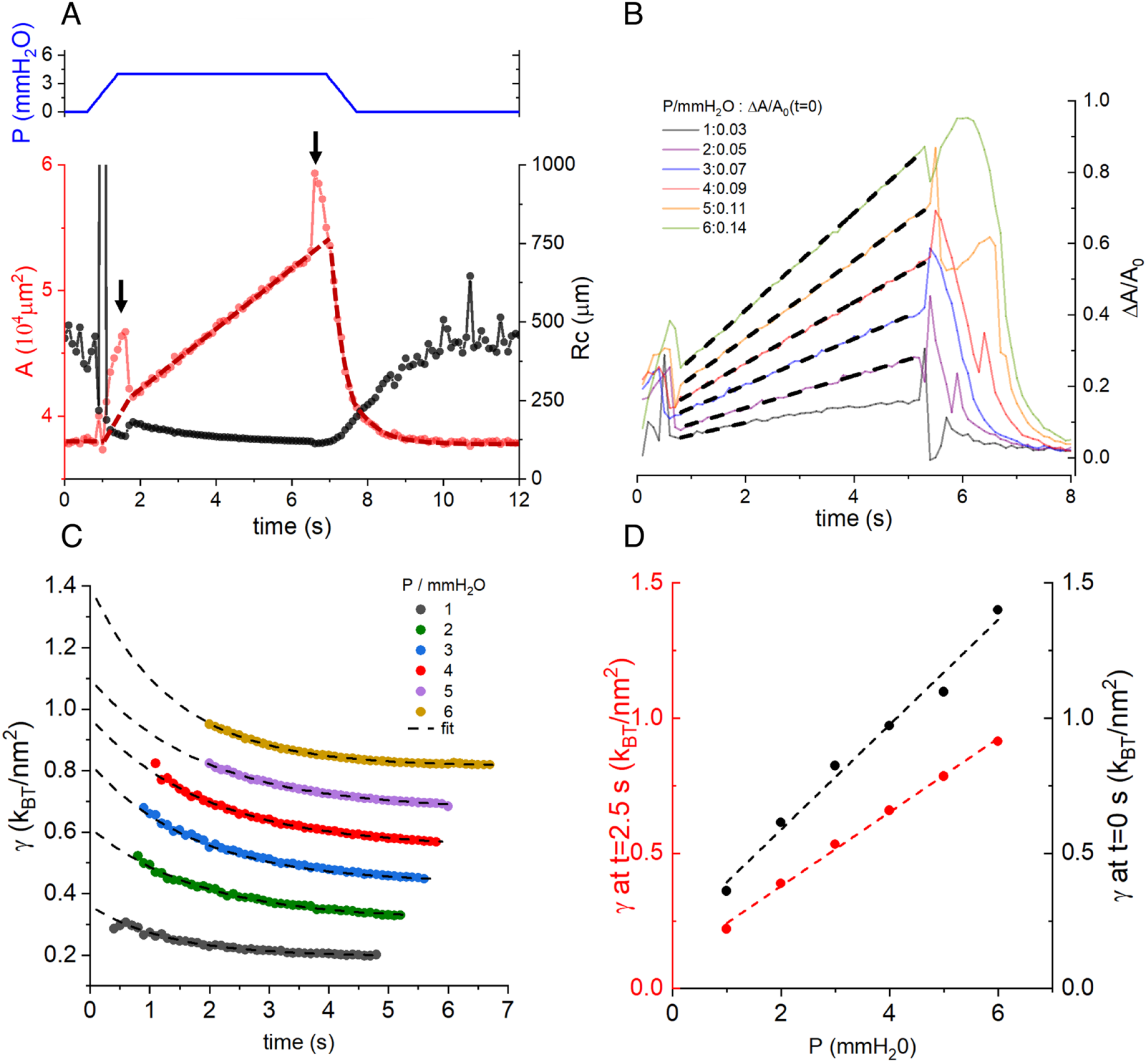
Measurements in nonequilibrium. (*A*) Expansion and contraction of a bilayer during the application of a pressure step. The area (red circles) and radius of curvature, Rc (black circles), of a bilayer as a function of time are shown for a POPE:POPG (3:1 wt:wt) bilayer during the application of the pressure pulse shown in blue (4 $mmH_{2}O$ ). Black arrows indicate the peak-like artifacts caused by the movement of the manometer. A dark red dashed line shows the (extrapolated) behavior of the area after removing the peaks. Two regimes of expansion can be seen, an initial fast expansion from 1 to 2 s followed by a slow expansion from 2 to 7 s before the pressure is removed and the bilayer contracts to its initial area. The Rc follows an opposite trend reflecting the increase in the curvature of the bilayer. (*B*) Relation between applied pressure and area expansion/contraction shown as relative changes in area ( $\frac{\Delta A}{A_{0}}$ ) as a function of time for the application of increasing pressure pulses from 1 to 6 $mmH_{2}O$ . Curves were aligned to the first peak artifact, e.g., black arrow in panel *A*. (*C*) Membrane tension ( γ ) for different pressures as a function of time (colored circles) calculated from data shown in panel *B*. After the application of pressure, a fast rise in the bilayer tension can be seen followed by a decay that can be fit with single exponentials (dotted lines, $r^{2}=0.99$ ). (*D*) Membrane tension at time zero (black), extrapolated from exponential fits, and at 2.5 s (red curve) as a function of pressure.

When there is a sudden application of pressure, sufficiently fast to overcome the relaxation of the freestanding bilayer (nonequilibrium regime), the observed behavior is different. Fig. 5*A* shows a typical result for a freestanding bilayer when a pressure step of 4 $mmH_{2}O$ was applied and held for 6 s before returning to zero pressure. The red curve shows the change in area measured by the capacitance method. During the application (or removal) of pressure, a spike artifact in the area occurred due to the movement of the manometer (black arrows). A dark-red dashed curve was drawn to approximate the expected shape of the area trace without these artifacts. During fast pressurization, the area of the bilayer increased monotonically with two regimes. Immediately after the application of pressure (at 1 s), the area increased rapidly for ~1 s, after which the rate decreased and persisted at a constant rate until the pressure was removed at about 7 s. We call these fast and slow expansion regimes, respectively. Fig. 5*B* shows the relative area change ( $\frac{\Delta A}{A_{0}}$ ) of a freestanding bilayer subjected to increasing pressure steps. To aid the visualization of the data, the traces were aligned to the spike artifact at the beginning of the stimulus. As can be seen, the slopes of both fast and slow regimes increased with pressure (Fig. 5*B*). The slow expansion of freestanding bilayer area, observed under the microscope as a persistent growth of bilayer area (Movie S1), continued until the pressure was removed or until the freestanding bilayer ruptured. Because of this “runaway” area growth, we performed all subsequent experiments while keeping the pressure pulses short (<6 s) to prevent significant deformation of the freestanding bilayer (Movie S2). *SI Appendix*, Fig. S3 shows later that specific capacitance does not change significantly for short applications of pressure.

We note that for low pressures ( $\leq 1.0\;mmH_{2}O$ ), the slow expansion ceases and approaches a finite area rather than runaway behavior (Fig. 5*B*, gray curve), consistent with the observation that low pressures ( $\leq 2.0-3.0\;mmH_{2}O$ ) are insufficient to promote runaway expansion of the freestanding bilayer, allowing instead a stable equilibrium condition, as shown in the previous section.

From the area curves and the optically measured radius, a , of each freestanding bilayer, we calculated $R_{c}$ as a function of time from Eq. **3** (Fig. 5*A*, black curve) (21). Then, substituting $R_{c}$ and the manometer pressure into Eq. **2**, we calculated the instantaneous bilayer tension as a function of time for pressures ranging from 1.0 to 6.0 $mmH_{2}O$ (Fig. 5*C*). Due to the artifacts introduced by the movement of the manometer, only values within the slow expansion regime were used for the calculation. Given that the manometer moves at a finite speed (~ 3 mm/s), it took longer to reach higher pressures, leading to a shift in the tension traces when aligned to time zero, the time just before pressure application (Fig. 5*C*). At these higher pressures, we observed a rapid initial increase in tension followed by a relaxation that decays to an increasingly higher minimum as larger pressures were applied. This relaxation follows a single exponential decay (r^2^ = 0.99), which we fit to the data to retrieve by extrapolation the value of tension at the instant of pressure application (t = 0). This would be the tension obtained if our manometer could apply pressure instantaneously and thus represents the highest tension value extractable from our system. We found that these tension limits follow a linear trend with applied pressures (Fig. 5*D*, black). This linear relationship between tension and pressure holds for arbitrary times, as shown in Fig. 5*D* for the tension measured at 2.5 s of pressurization.

### TRAAK Activity as a Function of Lateral Membrane Tension.

TRAAK is a stretch-activated potassium channel found in mammalian nodes of Ranvier, the booster stations of action potential propagation in myelinated nerve fibers (25, 26). When analyzed in GUVs or in cells in which the channel is expressed, TRAAK is active at baseline (i.e., in the absence of external stimulation) but can be activated further by pressurizing excised patches (11). Given that the tension-dependence of TRAAK gating has never been quantified, we applied the bilayer tensiometer to analyze TRAAK’s tension-dependent gating.

**Fig. 6.**
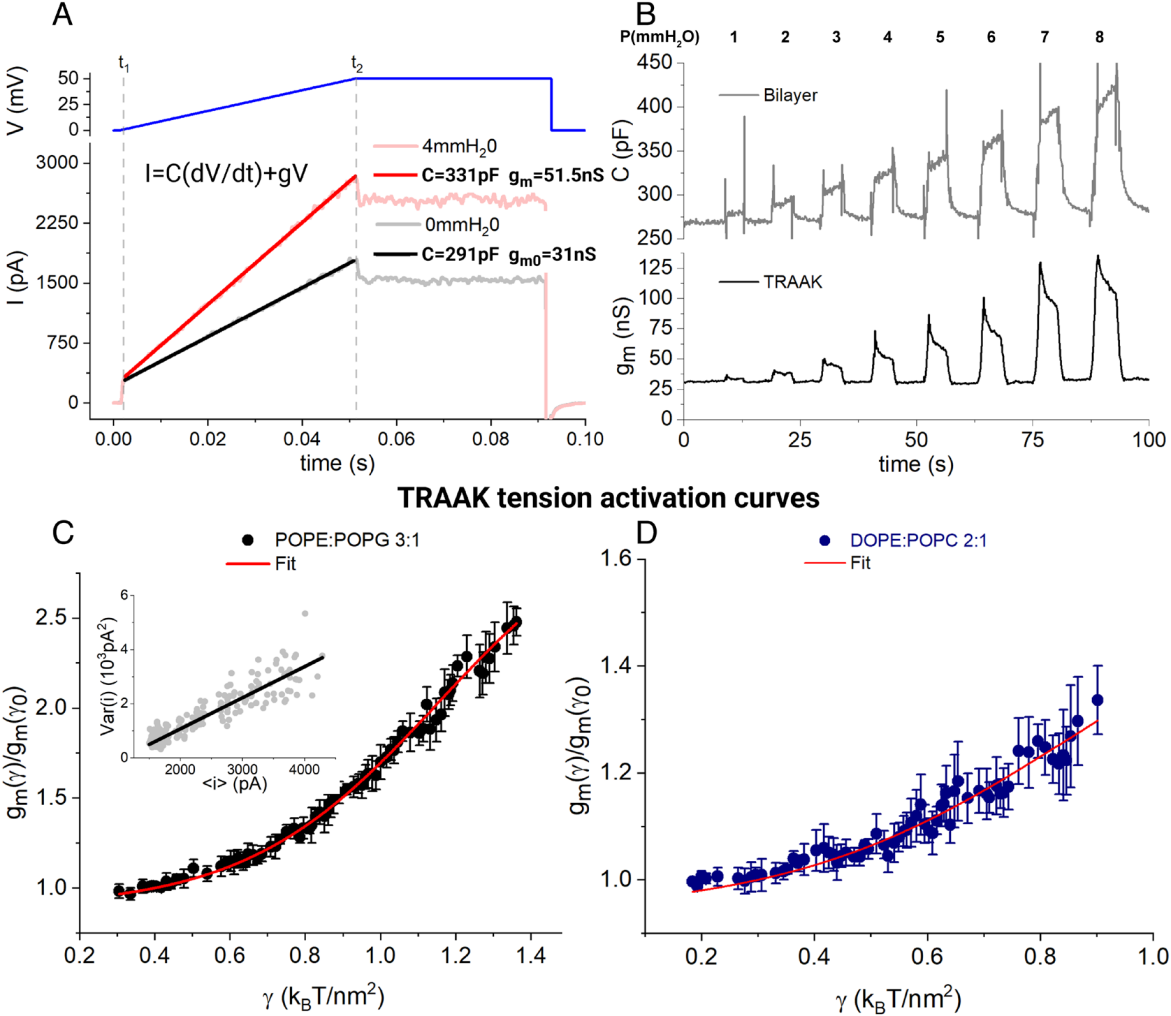
TRAAK activity as a function of bilayer tension. (*A*) Example of an experimental time point (100 ms long) for a POPE:POPG (3:1) bilayer with TRAAK channels. The voltage protocol (V) applied is shown in blue and measured currents (I) at zero-pressure and during a 4-$mmH_{2}O$ pressure pulse are shown in gray and red, respectively. Linear fits between times t_1_ and t_2_ are also shown in dark colors from where the capacitance (C) and the conductance (g_m_) can be obtained using the equation shown, from the intersection and slope, respectively. g_m0_ stands for the basal macroscopic conductance of TRAAK (P = 0), and g_m_ stands for its conductance at P > 0. By successively measuring these time points during the application of increasing pressures, time courses are obtained (*B*) for the capacitance (area) of the bilayer (*Top*, gray) and the conductance of TRAAK (*Bottom*, black). Capacitance measurements are then converted into lateral tension ( γ ) and correlated to TRAAK conductance at every time point. *Tension activation curves* (*C* and *D*) are plotted as TRAAK relative change in conductance from a reference state ( $g_{m}\left(\gamma \right)/g_{m}\left(\gamma_{0}\right)$ ) as a function of the bilayer tension ( γ ) for POPE:POPG (3:1) (n = 4) (*C*) and DOPE:POPC (2:1) (*D*) bilayers (n = 5). Reference states ( $g_{m}\left(\gamma_{0}\right)$ ) are $g_{m}\left(0.4\;\frac{k_{B}T}{{\mathrm{nm}}^{2}}\right)$ and $g_{m}\left(0.3\;\frac{k_{B}T}{{\mathrm{nm}}^{2}}\right)$ for POPE:POPG and DOPE:POPC bilayers, respectively. *Inset* in figure *C* shows a variance vs. mean plot for a single experiment. Fitting of the data with our gating model yielded the values $A=4.0\pm 0.2\;{\mathrm{nm}}^{2}$, $\begin{array}{c} \Delta G_{0}=4.5\pm 0.1\;k_{B}T,\;g_{l}/\left(Ng_{u}\right)=0.39\;\pm \;0.03 \end{array}$ , for POPE:POPG bilayers (r^2^ = 0.99) and $A=4.0\pm 1.5\;{\mathrm{nm}}^{2}$, $\Delta G_{0}=3.4\;\pm \;0.5\;k_{B}T$, $g_{l}/\left(Ng_{u}\right)=1.4\;\pm \;0.8$ , for DOPE:POPC bilayers (r^2^ = 0.94).

Channels were reconstituted into proteoliposomes consisting of 1-palmitoyl-2-oleoyl-sn-glycero-3-phosphoethanolamine (POPE):1-palmitoyl-2-oleoyl-sn-glycero-3-phospho-(1′-rac-glycerol) (POPG) (3:1) at a 1:100 (wt:wt) protein-to-lipid ratio and fused with freestanding bilayers of the same lipid composition using an established protocol (27). To measure the bilayer’s capacitance and the channel’s conductance simultaneously, we used a modification of the capacitance protocol described above. We applied successive sweeps of a voltage stimulus consisting of an initial ramp from 0 to 50 mV over 50 ms, followed by a constant voltage interval of 50 mV for 40 ms, before returning to a holding voltage of 0 mV (Fig. 6*A*). The current I across the freestanding bilayer at any point in time is given by $I=C\frac{\mathrm{dV}}{\mathrm{dt}}+g\;V$ , where C is the capacitance and V the voltage. Note that the voltage ramp between $t_{1}$ and $t_{2}$ (Fig. 6*A*, blue line) caused the current to increase with an initial abrupt current step followed by a smooth, linear increase (Fig. 6*A*, black line). The abrupt step in current at the beginning of the ramp corresponds to the $C\frac{\mathrm{dV}}{\mathrm{dt}}$ term, because $\frac{\mathrm{dV}}{\mathrm{dt}}$ was switched from 0 to a finite, constant value. The linear increase in current during the ramp corresponds to the g V term, proportional to V . Therefore, the capacitance is derivable from the abrupt current step and the conductance is equal to the slope of the linear increase in current. The conductance originates in K^+^ ions crossing the membrane through the TRAAK channels. To appreciate this, compare the current response to a voltage ramp recorded in the absence of channels, in which case the slope following the initial step in current is approximately zero (Fig. 3*A*, black trace), to the current response in the presence of TRAAK channels, in which case the slope is positive (Fig. 6*A*, black trace).

When the voltage stimulus was applied to the same freestanding bilayer with a pressure difference across it, both the current step (proportional to the capacitance) and the slope (equal to the conductance) increased (Fig. 6*A*, red trace). We repeated the voltage stimulus, each time measuring the capacitance and conductance while applying different pressures across the membrane for 6-sec intervals, returning the membrane to zero pressure in between. The capacitance and conductance from each voltage stimulus were graphed as a function of time (Fig. 6*B*). Following each pressure step, the capacitance gradually increased as the freestanding bilayer area increased as described earlier (Fig. 5*A*). At the same time, after an initial increase, the conductance gradually decreased, akin to the decrease in tension γ following a pressure step (Fig. 5*C*). Using the method described above, we converted freestanding bilayer capacitance to $R_{C}$ , which, with the applied pressure ΔP , was converted to γ through Eq. **2**.

In order to compare results from different experiments, we calculated the conductance relative to a reference value for each experiment. We then plotted the mean value of the relative conductance (±SD) over all experiments as a function of tension (*Methods* and Fig. 6 *C* and *D*). The curves show the relative change in TRAAK conductance as a function of tension. Reference values were selected as the lower values of tension that are both explicitly measured and shared by all the experiments.

The conductance increase is gradual, unlike tension activation of mechanosensitive channels MscL and Piezo1 (13, 28, 29). In TRAAK, a maximum conductance is not approached even while tension is increased to values corresponding to the half lytic tension (~3.5 $\frac{k_{B}T}{{\mathrm{nm}}^{2}}$ ) of lipid bilayers. Experiments using bilayers consisting of DOPE:POPC (2:1) gave similar results, but we note that tension values obtained for a given pressure value were smaller in DOPE:POPC (2:1) and that higher tensions were achievable in POPE:POPG (3:1) (Fig. 6*D*).

TRAAK currents were recorded at 50 mV during the 40 msec interval after the voltage ramp (Fig. 6*A*). With applied lateral membrane tension the mean current increased from less than 2,000 pA to greater than 4,000 pA (Fig. 6 *C*, *Inset*). Notably, the current variance increased as a linear function of the mean current. This increase indicates that the TRAAK open probability, even at the highest applied tensions, is less than 0.5, and the linear relationship suggests that the open probability is low (30). Thus, in these experiments, TRAAK channels are already open to some degree at baseline tension, 0.2 to 0.3 $\frac{k_{B}T}{{\mathrm{nm}}^{2}}$ , are further activated with a weak dependence on tension, and never reach high open probability even at relatively high values of lateral membrane tension.

**Fig. 7.**
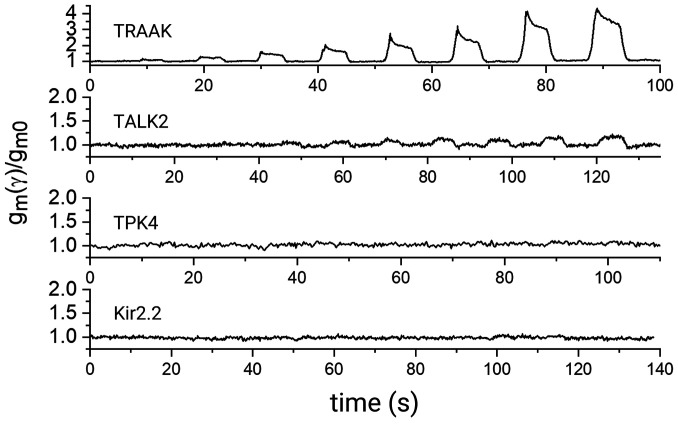
Mechanical response of different potassium channels. Fold-activation time courses are shown as the relative increase in conductance during incremental pulses of pressure from the unpressurized state ( $g_{m}\left(\gamma \right)/g_{m0}$ ), for TRAAK, TALK2, TPK4, and Kir2.2. $g_{m0}$ is the macroscopic conductance of the channel at zero-pressure.

We show three additional K^+^ channels analyzed using the bilayer tensiometer (Fig. 7 and *SI Appendix*, Fig. S2). TALK2 exhibited a small response to tension, up to a 15% increase above baseline activity at the highest tensions, while Kir2.2, a mammalian inward rectifier K^+^ channel (31), and TPK4, a plant K^+^ channel (32), exhibited no detectable response to changes in membrane tension over the tension range studied.

### Decoupling Membrane Curvature and Tension as the Mechanosensory Gating Stimulus.

**Fig. 8.**
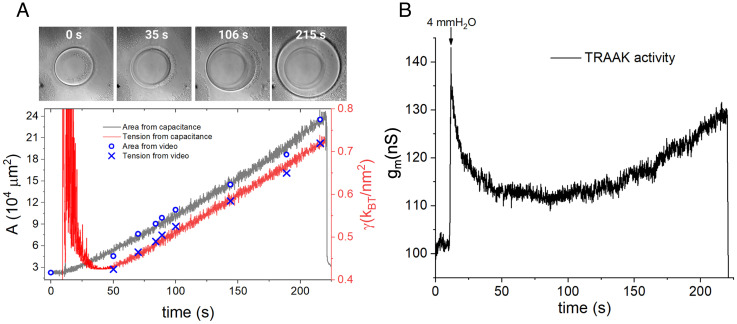
TRAAK and limits of nonequilibrium measurements. (*A*, *Top*) Frames from a video showing the expansion of a DOPE:POPC 2:1 (wt:wt) bilayer during the application of a long pulse of 4 $mmH_{2}O$ . (*Bottom*) values of tension, γ (blue x), and area, A (blue empty circles), calculated from radii of curvature measured directly from the frames of the video, and area (gray line) and tension (red line) calculated from capacitance measurements with a corrected Cs. After the initial relaxation, the tension increases linearly during the expansion of the bilayer. (*B*) TRAAK conductance ( $g_{m}$ ) as a function of time during the bilayer expansion. A black arrow points to the moment when the pressure (4 $mmH_{2}O$ ) is applied. The conductance shows a minimum closely correlated with the occurrence of the maximum curvature of the bilayer.

Eq. **2** tells us that for a pressurized bilayer, γ is directly proportional to the product $\Delta P\;R_{C}$^**^. As described above, we observed that following an increase in ΔP , the membrane area slowly increases as lipid molecules move from the torus to the bilayer, and thus, $R_{C}$ decreases. This is the origin of the gradually diminishing tension following the initial increase after a pressure step. After sufficient time, $R_{C}$ will decrease to a minimum when the membrane becomes a hemisphere, at which point its radius of curvature (Rc) equals the hole radius. If the membrane is permitted to expand beyond a hemisphere, like a bubble extending outside the hole, then $R_{C}$ will increase and, since ΔP remains constant, γ will also increase. Fig. 8*A* shows the monotonic increase in freestanding bilayer area up to and beyond the formation of a hemisphere, along with the lateral membrane tension calculated through Eq. **2**, which exhibits a minimum. Fig. 8*B* shows the conductance of TRAAK channels during such a prolonged expansion. As one would anticipate if TRAAK is opened by lateral membrane tension, the conductance also exhibits a minimum. This result precludes a model in which TRAAK is activated by membrane curvature, i.e., proportional to ( $\frac{1}{R_{C}}$ ), instead of tension (33). If membrane curvature were the primary stimulus to open the channel, then conductance would exhibit a maximum rather than a minimum when the freestanding bilayer forms a hemisphere. This conclusion holds even if the TRAAK channels are reconstituted with an orientation in both directions in the membrane. Therefore, the primary mechanical stimulus for TRAAK activation is lateral membrane tension.

## Discussion

Many studies of mechanosensitive ion channels utilize pressure as the stimulus variable because it is easy to know the pressure in an experiment. But if the channel responds to lateral membrane tension, which appears to be the case for numerous mechanosensitive channels, both the pressure and the radius of curvature must be known. Suction pipettes on GUVs allow accurate measurements of membrane curvature (34), but it is very difficult to record ion channel activity in whole GUVs (18). Patch recording methods provide membranes with channels in them that can be recorded; however, it is difficult to measure the radius of curvature with accuracy in patch pipettes. For this reason, we built a lipid bilayer tensiometer. The method, like patch pipette pressurization, involves measuring membrane curvature to calculate tension using the Young–Laplace equation. The membrane curvature can be measured with much higher accuracy than in patch pipettes and therefore, the tensiometer allows the characterization of channel activity with better control of lateral membrane tension.

The tensiometer relies on and extends past studies of planar lipid bilayer rheology and mechanics (35–37). Zero-pressure tension sets the lower limit, and rapid pressurization in the setting of slow mechanical relaxation sets the upper limit, rendering the device useful for studying lateral membrane tensions in the range 0.2 to 1.4 $\frac{k_{B}T}{{\mathrm{nm}}^{2}}$ . This range is relevant for many mechanosensitive ion channels, including MscS, MscL, TRAAK, and possibly Piezo1 (3, 13, 38, 39). Some ion channels with mechanical sensitivity, such as certain voltage-dependent K^+^ channels, operate below this sensitivity range and thus cannot be analyzed with the device (40). The optics and laser illumination in the device we have built far exceed what is necessary for others to implement, but we needed these features initially for optical mapping to learn under what conditions electrical capacitance can be used to calculate membrane curvature. The basic hardware requirements for future implementation thus include a patch clamp amplifier, horizontal bilayer chamber, manometer, widefield microscope, and the ability to produce round holes in plastic by a simple technique described in the methods.

We applied the tensiometer to quantify the tension sensitivity of the TRAAK K^+^ channel. We show that as the membrane geometry changes under constant pressure, a conductance minimum occurs under the condition of maximum curvature and minimum tension (Fig. 8). Thus, TRAAK gates open in response to lateral membrane tension, not membrane curvature. We also find that TRAAK channels are open at the smallest tensions achievable, ~0.2 $\frac{k_{B}T}{{\mathrm{nm}}^{2}}$ , and would still be open even at zero tension (Fig. 6 *C* and *D*). This finding is consistent with demonstrations that TRAAK channels exhibit ion conduction in unstimulated whole-cell recordings and nodes of Ranvier, both of which are presumably conditions of near-zero lateral membrane tension (11, 26). TRAAK conductance gradually increases as tension is increased over the entire range ~0.2 to 1.4 $\frac{k_{B}T}{{\mathrm{nm}}^{2}}$ . The upper limit of this range is about half the lytic tension of lipid bilayers, high for biological processes. For comparison, in cell-attached recordings, the Piezo1 ion channel opens nearer the lower end of this tension range and more steeply (13). The bacterial mechanosensitive channel MscL opens nearer the higher end of the range, compatible with its role as a high-tension sensor to prevent osmotic explosion, but like Piezo1, it opens as a steep function of tension. Thus, TRAAK is unique because it begins to open at small tensions but continues to open gradually as tension is increased. Using nonstationary noise analysis, we show that TRAAK’s open probability is less than 0.5 even at the highest tensions applied (Fig. 6*C*). This low open probability is consistent with the shape of the conductance–tension curves, which do not approach a maximum conductance level (Fig. 6 *C* and *D*).

TRAAK’s sensitivity to tension is reflected in the curvature or steepness of the relationship between conductance and tension. To quantify the sensitivity without appealing to a specific physical model for its origin, we expressed the free energy for channel opening as a power series in γ to first order (*Methods* and Fig. 6 *C* and *D*). The coefficient for the first order term, ~4 *nm*^2^, describes the sensitivity. The corresponding value for MscL is ~$20\;{\mathrm{nm}}^{2}$ (29, 41). Thus, TRAAK has about one-fifth the tension sensitivity of MscL. Whether this weak sensitivity that can modulate TRAAK conductance over a wide range of tensions is important to its biological function is still unknown, mainly because we do not have a good estimation of the range of tensions experienced by the nodes of Ranvier. As we proposed previously (25), it is possible that TRAAK channels in nodes could be a protective mechanism against mechanically induced ectopic action potentials. It is even possible that they could play a role in repolarization of action potentials through coupling of membrane depolarization to associated mechanical changes in the node.

In summary, this paper presents the design and construction of a bilayer tensiometer for the accurate study of mechanosensitive channels that respond to lateral membrane tension. We apply the device to quantify tension-dependent gating in the TRAAK K^+^ channel.

## Materials and Methods

### Protein Expression and Purification.

TRAAK from *Homo sapiens* (42) was cloned into a pEG BacMam vector (43). At the C-terminus of the construct, a protease cleavage site (PreScission), an enhanced green fluorescent protein (eGFP), and a 10-Histidine tag were placed for purification. For overexpression and protein purification, HEK-293S GnTl-cells were grown in suspension at 37 °C and then infected with P3 BacMam virus of the TRAAK construct at a density of ~3 × 10^6^ cells/mL. At 8 to 12 h postinfection, 10 mM sodium butyrate was added to the culture and the temperature was lowered to 29 °C. Cells were harvested by centrifugation 48 h after infection, frozen in liquid N_2_, and stored at −80 °C until needed. Frozen cells were thawed and solubilized in 50 mM Tris pH 8.0, 150 mM KCl, 1 mM ethylenediaminetetraacetic acid (EDTA), 2.0% (wt:vol) n-dodecyl-b-D-maltopyranoside (DDM), and a protease inhibitor cocktail (0.1 mg/mL pepstatin, 1 mg/mL leupeptin, 1 mg/mL aprotinin, 0.1 mg/mL soy trypsin inhibitor, 1 mM benzamidine, and 1 mM phenylmethylsulfonyl fluoride). After 1.5 h of solubilization, lysed cells were centrifuged at 36,000 g for 30 min and the supernatant was incubated with GFP nanobody affinity resin (1 mL of resin per liter of culture) for 1 h at 4 °C with gentle mixing. The resin was loaded onto a column and washed with buffer A (20 mM Tris-HCl pH 8.0, 150 mM KCl, 1 mM EDTA, 6 mM DDM). eGFP and affinity tags were cut with PreScission protease overnight at 4 °C. The cleaved protein was then concentrated to a volume of 1 mL and run on a Superdex 200 10/300 GL gel filtration (GE Healthcare) column equilibrated in 20 mM Tris-HCl pH 8.0, 150 mM KCl, 1 mM EDTA, and 1 mM DDM.

*TPK4* (full length) from *Arabidopsis thaliana* was expressed and purified as described above for *TRAAK*, with the following exceptions: instead of DDM, n-decyl-b-D-maltopyranoside DM was used for extraction (2.0%), wash, and size exclusion (0.2%), and instead of Tris-HCl, 10mM potassium phosphate buffer and 5 mM DTT were used throughout the purification.

*TALK2* from *Oreochromis niloticus* was expressed in *Pichia Pastoris* as previously reported for other proteins (31, 44) and purified as described above for *TRAAK*.

*Kir2.2* from *Gallus gallus* was expressed and purified as previously reported (31).

### Reconstitution of Purified Proteins into Liposomes.

A mixture of lipids composed of 3:1 (wt:wt) 1-palmitoyl-2-oleoyl-sn-glycero-3-phosphoethanolamine (POPE, Avanti Polar Lipids): 1-palmitoyl-2-oleoyl-sn-glycero-3-phospho-(1′-rac-glycerol) (POPG, Avanti Polar Lipids) was used for the reconstitution of TRAAK, TALK2, Kir2.2, and TPK4 channels into lipid vesicles. (44, 45) The above lipid mixture in chloroform was dried under an argon steam followed by vacuum for ~2 h and resuspended at a concentration of 20 mg/mL in reconstitution buffer (10 mM Hepes pH 7.4, 150 mM KCl). The lipid suspension was dispersed to clarity by sonication in a bath sonicator (CPX1800H, Branson) and solubilized with 1.0% (wt:vol) DM. TRAAK, Kir2.2, and TPK4 were diluted with reconstitution buffer supplemented with 0.2% (wt:vol) DM to 0.2 mg/mL (∼51 µM). For TALK2, lipids were solubilized with 4.0 % (wt:vol) n-octyl-b-D-maltopyranoside. Equal volumes of 20 mg/mL solubilized lipid mixture and each of these protein solutions were combined to make protein:lipid (wt:wt) ratios of 1:30 (TALK2), 1:10 (Kir2.2), 1:100 (TRAAK), and 1:60 (TPK4) with a final lipid concentration of 10 mg/mL. For TRAAK, TALK2, and TPK4, the detergent was removed by dialysis against reconstitution buffer at 4 °C for 2 to 4 d. For TALK2, the mixture was further incubated in bio-beads (Bio-Rad) for 1 d. Proteoliposomes were then frozen in liquid N_2_ in aliquots of 20 µL and stored at −80 °C until used. For Kir2.2 and TPK4, 5 mM DTT was added to the dialysis buffer. For Kir2.2, the dialysis was stopped after 24 h and the protein was incubated with bio-beads (Bio-Rad) at 4 °C for another 24 h. Just before an experiment, proteoliposomes were thawed and 3 M KCl was added to a concentration of 450 mM followed by brief sonication in a bath sonicator.

### Bilayer Tensiometer Setup.

The microscope used for optical recordings consists of an FN1 upright microscope (Nikon) connected to an ORCA flash V4 sCMOS camera (Hamamatsu) controlled by a computer through the software NISelements 5.2 (Nikon). To observe the bilayers by transmitted light, a white-light LED was added to the recording chamber. To observe bilayers by fluorescence, a custom-made laser illumination system was implemented that consisted of a 532-nm GEM laser (Laser Quantum), expanded and fed into a LSKGG4/M Galvo-Galvo scanner head (Thorlabs), henceforth termed GG, followed by an Apodization mask, AP (Photo-Sciences), (45) and a TL20X-MPL objective, XO (Thorlabs) used to focus the laser on the bilayer. The optical planes of the GG, AM, and XO were conjugated by relay lenses (Thorlabs). Custom-made software (Labview) was used to control the GG by computer through a BNC-2110 Terminal Block (National Instruments). All the experiments were carried out using a CFI60 Nikon 40× (0.8 NA) objective, DO.

Pressures across the bilayers were applied by a manometer consisting of a 50-mL syringe held by a MP-285/M micromanipulator (Sutter). The syringe was connected to the bottom of the recording chamber. The manipulator was controlled both manually and by a computer through an MPC-200 and a ROE-200 controller (Sutter). Another manipulator connected to the same MPC-200 unit was used to move the recording chamber.

For optical recordings at equilibrium, pressure levels were reached manually by controlling the manometer at a rate of ~10 µm/s, waiting 2 to 5 min for stabilization of the bilayer. For recordings in the nonequilibrium regime, pressure levels and protocols were preset and performed automatically using the software Sutter Multi-Link Position Control.

Bilayer heights (normal to the axis of the DO) were obtained by focusing the microscope on an illuminated point on the bilayer and then reading the displacement of the DO with a 542-158 LGK 10-mm linear gauge (Mitutoyo) attached to the body of the microscope through a custom-made adaptor. Values were read directly from an EH-10P Multi-Function Single Display (Mitutoyo).

### Planar Bilayer Experiments.

Partitions for recordings were prepared by making a small indentation at the center of 2 × 1.5-cm FEP (fluorinated ethylene propylene copolymer, 100-µm thick, Goodfellow) films with a tungsten needle (Fine Science Tools), followed by an electric discharge across the indentation with a BD10-AS high-frequency generator (Electro-Technic Products). Partitions were then sonicated and stored in ethanol until use. Before use, a small amount (~0.5 µL) of 10 mg/mL lipid solution in decane composed of either 2:1 (wt:wt) of 1,2-dioleoyl-sn-glycero-3-phosphoethanolamine (DOPE, Avanti Polar Lipids):1-palmitoyl-2-oleoyl-sn-glycero-3-phosphocholine (POPC, Avanti Polar Lipids) or 3:1 1-palmitoyl-2-oleoyl-sn-glycero-3-phosphoethanolamine (POPE, Avanti Polar Lipids):1-palmitoyl-2-oleoyl-sn-glycero-3-phospho-(1′-rac-glycerol) (POPG, Avanti Polar Lipids) was smeared over the electro-formed hole and allowed to air dry for 10 to 30 min.

The partitions were assembled separating two chambers in a 3D-printed sample holder (VisiJet M3 Crystal, ProJet MJP 3600) designed for these experiments. The same buffer (10 mM potassium phosphate pH 7.4, 100 mM KCl) was used in both chambers. After waiting ~5 min to allow for the equilibration of pressure across the system, <0.2 µL of 10 mg/mL of lipid solution in decane was added (with a pipette tip) over the hole to form a bilayer. Immediately after the addition of lipid, an air bubble was dragged through the hole to disperse the lipid solution. The formation of the bilayer was confirmed both optically and electrically before proceeding. For electrical recordings, the voltage across the lipid bilayer was clamped with an Axopatch 200B amplifier in whole-cell mode. The analog current signal was low-pass filtered at 1 kHz (Bessel) and digitized at 10 kHz (Digidata 1550B digitizer, Molecular Devices). Digitized data were recorded on a computer using the software pClamp (Molecular Devices). Recordings were performed at room temperature.

### Short-Pressure Pulse Experiments.

After forming a lipid bilayer, the zero-pressure point was found (*Results* and Fig. 1*C*). The pressure protocol was set by manually bringing the manometer to different heights relative to the zero-pressure point, covering a range of 1 to 8 $mmH_{2}O$ in steps of 1 mm, and then recording these positions on the Multi-Link Position Control software (Sutter). A protocol was then written in the Position Control software in which each recorded height was held for 6 s before returning to the zero-pressure point height for another 6 to 8 s. Proteoliposomes containing TRAAK, TALK2, Kir2.2, or TPK4 at protein:lipid ratios (wt:wt) 1:100, 1:10, 1:10, and 1:60, respectively, were applied to the bilayer and allowed to settle for ~2 min followed by the addition of 1 µL of 3M KCl to promote their fusion to the bilayer. After channel fusion, the pressure protocol was applied automatically using the Position Control software.

### Long-Pressure Pulse Experiments.

After channel fusion a pressure of 4 $mmH_{2}O$ was applied using the Multi-link Position Control software as explained above, with the difference that the set height on the protocol (4 mm) was held for 210 s before returning to the zero-pressure point. The radius of curvature of the bilayer was measured directly from the frames of a timelapse video (Fig. 8*A*) by fitting a circle on the perimeter of the expanded bilayer using the NISelements software. These measurements were done after the perimeter of the bilayer's projection exceeded the perimeter defined by the hole in the partition (Fig. 8*A*, at 106 s). The area of the bilayer was calculated from the R_c_ and the size of the hole supporting the bilayer (Fig. 8*A* blue marks) (21). Using these area values and the measured bilayer capacitances, specific capacitances, Cs, were calculated throughout the expansion (*SI Appendix*, Fig. S3). The Cs as a function of time was plotted and fitted to a single exponential decay (r^2^ = 0.99) (*SI Appendix*, Fig. S3) with a time constant of 43 s. Therefore, if the pulses of pressure are within 6 s, the accumulated error on the Cs would be less than 7%, thus justifying the use of the Cs at the zero-pressure point for area calculation throughout short-pulse experiments. For long-pulse experiments (more than 6 s pulses), the fitted Cs(t) function should be used as a transfer function between the capacitance and the area.

### Data Analysis.

Values for capacitance and channel conductance were obtained from data recorded using the software Clampex and further processed with OriginPro (OriginLab). Data points shown in Fig. 6 *C* and *D* were obtained by first calculating the values of conductance relative to a reference state, pooling these values from different experiments and calculating the mean and SD every 10 points. This corresponds to binning the data every ~0.01 $\frac{k_{B}T}{{\mathrm{nm}}^{2}}$ and calculating the statistics on every bin. Reference points were chosen as the minimum value of tension that is both explicitly measured and shared by all the experiments as explained in the results section. Curve fits were performed with OriginPro.

### Model of Channel Gating.

TRAAK is assumed to adopt only two conformations, closed and opened, with probabilities $P_{c}$ and $P_{o}$ , respectively. If the energy difference between these conformations is expressed as,

[4] $$\Delta G\left(\gamma \right)={\Delta G}_{0}-A\gamma ,$$

with ${\Delta G}_{0}$ the energy difference when γ = 0 and A a coefficient for tension dependence (i.e., the linear term in a power series expansion of ΔG(γ) ), then,

[5] $$P_{o}\left(\gamma \right)=\frac{1}{1+\exp\left[\frac{\left({\Delta G}_{0}-A\gamma \right)}{k_{B}T}\right]}.$$

If a membrane contains N channels each with unitary conductance $g_{u}$ , then membrane conductance is

[6] $$g_{m}\left(\gamma \right)=N\;P_{O}\left(\gamma \right)\;g_{u}+g_{l},$$

with $g_{l}$ being a “leak” conductance. To combine data from multiple bilayers, $g_{m}\left(\gamma_{o}\right)$ was measured at a reference $\gamma_{o}$ common to all membranes and the quantity $\frac{g_{m}\left(\gamma \right)}{g_{m}\left(\gamma_{o}\right)}$ was graphed (Fig. 6 *C* and *D*). This analysis is valid if $g_{l}$ is proportional to $N\;g_{u}$ , which appears to be the case (*SI Appendix*, Fig. S4). This proportionality implies that the leak conductance is mediated by a fixed proportion of TRAAK channels in the membrane.

## Supplementary Material

Appendix 01 (PDF)

Movie S1. Timelapse recording showing a bilayer expanding after the sudden application of a pressure of 4 *mmH*_2_*O*. The playing speed was accelerated 20 folds.

Movie S2. Timelapse recording showing a bilayer subjected to pressure pulses of increasing magnitude from 1 to 5 *mmH*_2_*O*.

## Data Availability

All study data are included in the article and/or *SI Appendix*.

## Footnotes

^*^In this paper we use the term “lateral membrane tension” and “tension” synonymously.

^**^When ∆P = 0, Rc = ∞ and therefore γ is undefined by the Young-Laplace equation under this singular condition (we deduced its value by extrapolation to be 0.2-0.3 kBT/nm2). At finite values of ∆P and Rc, Eq. (**2**) applies.
