# Supplementary material for: Freestanding lipid bilayer tensiometer for the study of mechanosensitive ion channels
Source: Proc Natl Acad Sci U S A. 2023 Mar 13;120(12):e2221541120. doi: 10.1073/pnas.2221541120 (PMC10041094; doi:10.1073/pnas.2221541120)
Supplement: Supplementary file 1 — Appendix 01 (PDF) [file pnas.2221541120.sapp.pdf]

Supporting Information for
Freestanding Lipid Bilayer Tensiometer for the study of mechanosensitive ion channels

Gonzalo Pérez-Mitta and Roderick MacKinnon*

Affiliations

Laboratory of Molecular Neurobiology and Biophysics, Howard Hughes Medical Institute, The Rockefeller

University, New York, United States.

*Correspondence to: Roderick MacKinnon (mackinn@rockefeller.edu).

This PDF file includes:

SI_Figures 1 to 4
Legends for Videos 1 and 2

Other supporting materials for this manuscript include the following:

Videos 1 and 2

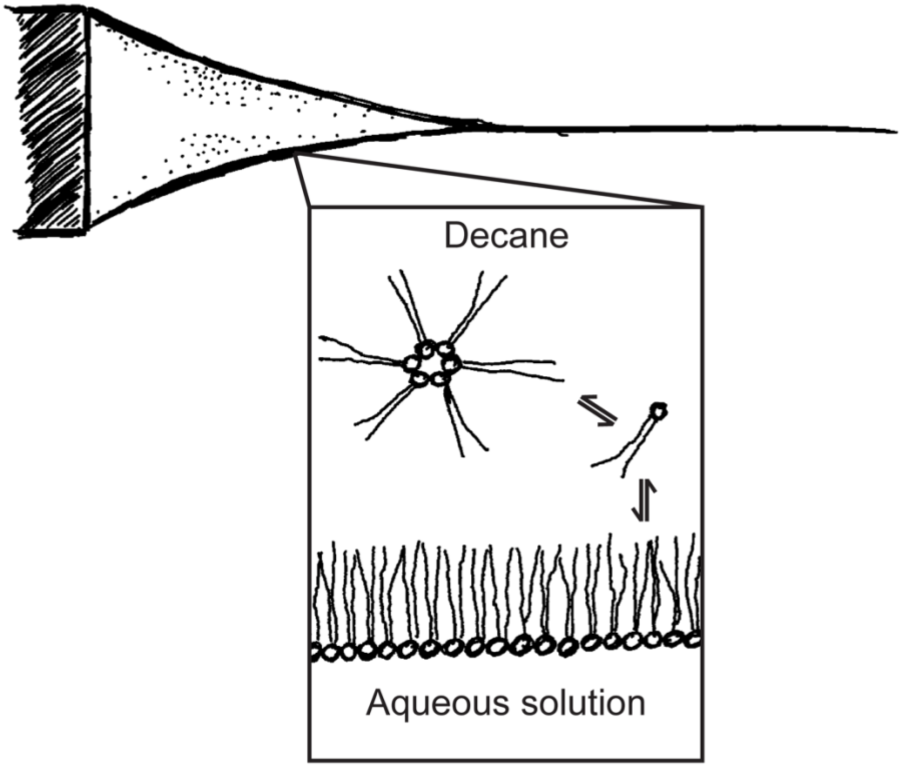

SI_Figure 1. Scheme of the cross-section of a black lipid membrane (BLM), showing the hypothesized molecular detail of a torus. Lipids are expected to form (reverse) micelles within the organic phase (decane) and a monolayer at the interface between the decane and aqueous solution. The contact of both opposing monolayers towards the center of the partition hole, gives rise to the lipid bilayer. The presence of the torus is likely responsible for the black lipid (freestanding) membrane mechanical behavior.

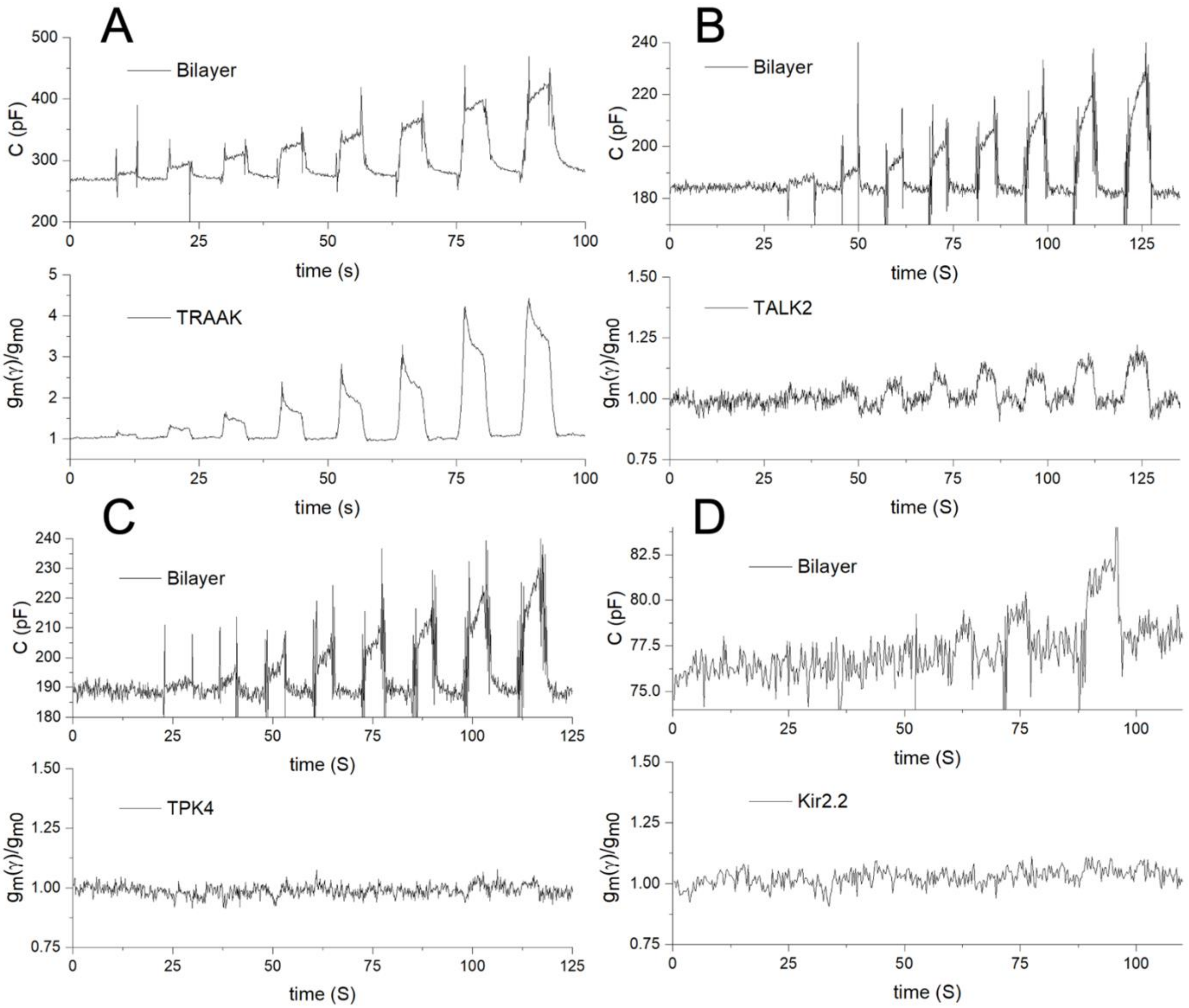

SI_Figure 2. Mechanical response of the bilayer and relative increase in conductance for the different potassium channels shown in figure 7. Capacitance (Top) and Fold-activation (Bottom), $g_m(\gamma)/g_{m0}$, for TRAAK (A), TALK2 (B), TPK4 (C), and Kir2.2 (D) during the application of incremental pulses of pressure.

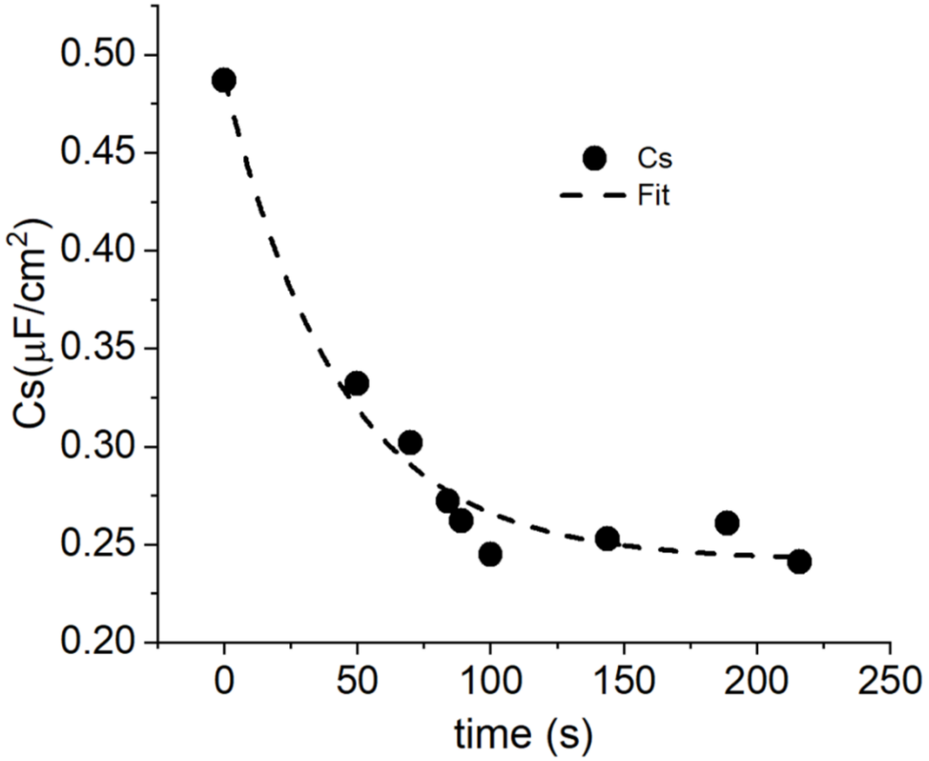

SI_Figure 3. Change in Cs as a function of time (black circles) and exponential fit (dotted line). The time constant of the fit is 43 sec, r^2=0.99.

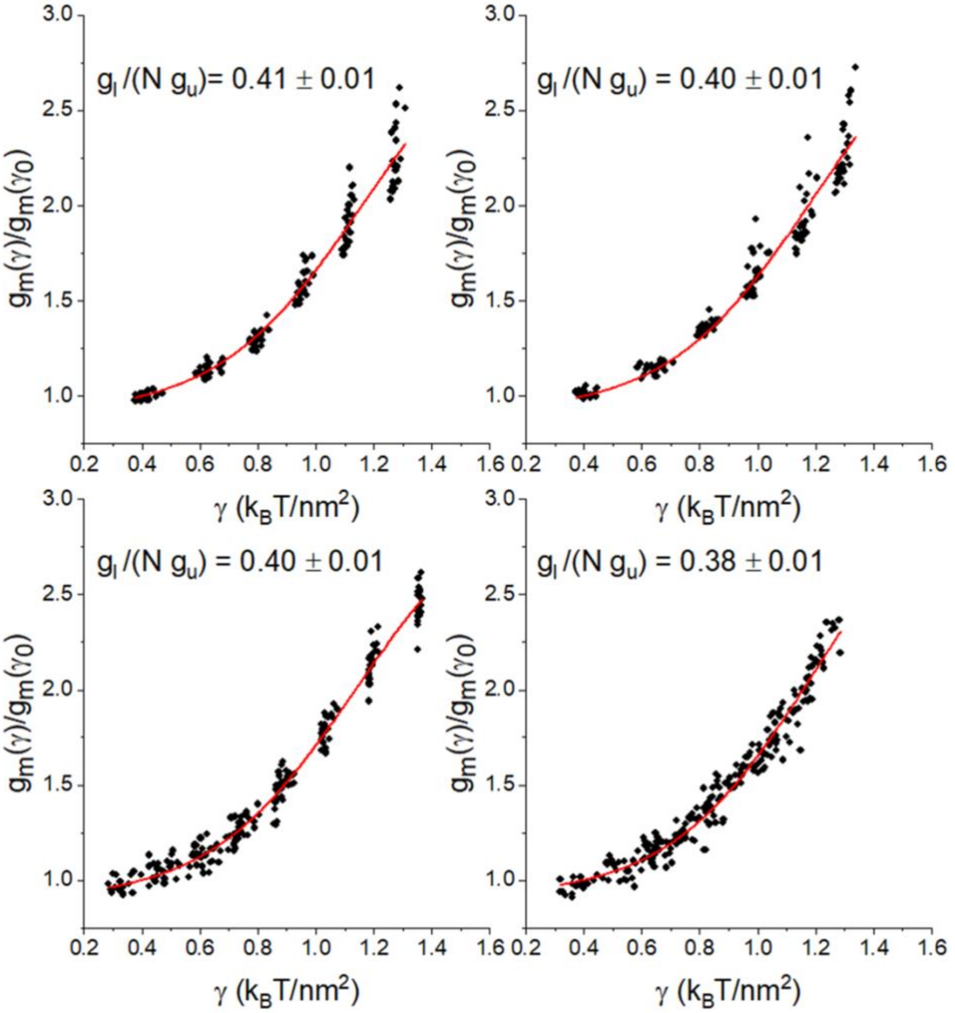

SI_Figure 4. TRAAK activity as a function of bilayer tension. Relative change in conductance of TRAAK (and fit) to the reference value at 0.4 $\frac{k_BT}{nm^2}$ for 4 different experiments. The fitted parameter $g_l/(Ng_u)$ is shown for each experiment.

Supporting video 1. Timelapse recording showing a bilayer expanding after the sudden application of a pressure of 4 mmH_2O. The playing speed was accelerated 20 folds.

Supporting video 2. Timelapse recording showing a bilayer subjected to pressure pulses of increasing magnitude from 1 to 5 mmH_2O.
